# Supplementary material for: Cloud BioLinux: pre-configured and on-demand bioinformatics computing for the genomics community
Source: BMC Bioinformatics. 2012 Mar 19;13:42. doi: 10.1186/1471-2105-13-42 (PMC3372431; doi:10.1186/1471-2105-13-42)
Supplement: Additional file 1 — Supplementary 1 Cloud BioLinux software documentation in the form of a mini, self-contained website. Users need to download and uncompress the .zip file, and open through a web browser the "index.html" file available on the main directory. (ZIP 1823 kb). [file 1471-2105-13-42-S1.ZIP › Cloud-BioLinux-Package-Documentation/docs/dnamlk.html]

Bio-Linux Software Documentation Pages

Back to search form

## dnamlk

|  |  |
| --- | --- |
| Name | dnamlk |
| Description | **dnamlk** is part of the PHYLIP package  Copyright 1986-2004 by the University of Washington. Written by Joseph Felsenstein. Permission is granted to copy this document provided that no fee is charged for it and that this copyright notice is not removed.  This program implements the maximum likelihood method for DNA sequences under the constraint that the trees estimated must be consistent with a molecular clock. The molecular clock is the assumption that the tips of the tree are all equidistant, in branch length, from its root. This program is indirectly related to DNAML. Details of the algorithm are not yet published, but many aspects of it are similar to DNAML, and these are published in the paper by Felsenstein and Churchill (1996).  The model of base substitution allows the expected frequencies of the four bases to be unequal, allows the expected frequencies of transitions and transversions to be unequal, and has several ways of allowing different rates of evolution at different sites.  **References:**  Felsenstein, J. 1993. PHYLIP (Phylogeny Inference Package) version 3.5c. Distributed by the author. Department of Genetics, University of Washington, Seattle.    Felsenstein, J. 1989. PHYLIP -- Phylogeny Inference Package (Version 3.2). Cladistics 5: 164-166. |
| Homepage | http://evolution.genetics.washington.edu/phylip.html |
| Remote Documentation | http://evolution.genetics.washington.edu/phylip/doc/dnamlk.html |
